# Experiences of patients with heart failure with medicines at transition intervention: Findings from the process evaluation of the Improving the Safety and Continuity of Medicines management at Transitions of care (ISCOMAT) programme

**DOI:** 10.1111/hex.13570

**Published:** 2022-07-31

**Authors:** Catherine Powell, Hanif Ismail, Maureen Davis, Andrew Taylor, Liz Breen, Beth Fylan, Sarah L. Alderson, Chris P. Gale, Ian Kellar, Jonathan Silcock, David P. Alldred

**Affiliations:** ^1^ School of Pharmacy and Medical Sciences University of Bradford Bradford UK; ^2^ Wolfson Centre for Applied Health Research Bradford UK; ^3^ Leeds Teaching Hospitals NHS Trust Research and Innovation Centre Leeds UK; ^4^ ISCOMAT Patient‐Led Steering Group University of Bradford Bradford UK; ^5^ NIHR Yorkshire and Humber Patient Safety Translational Research Centre Bradford Institute for Health Research Bradford UK; ^6^ School of Medicine University of Leeds Leeds UK; ^7^ Department of Cardiology Leeds Teaching Hospitals NHS Trust Leeds UK; ^8^ Leeds Institute for Data Analytics University of Leeds Leeds UK; ^9^ Leeds Institute of Cardiovascular and Metabolic Medicine University of Leeds Leeds UK; ^10^ School of Psychology University of Leeds Leeds UK; ^11^ School of Healthcare University of Leeds Leeds UK

**Keywords:** heart failure, medicines, process evaluation, qualitative, transitions

## Abstract

**Background:**

Medicines are often suboptimally managed for heart failure patients across the transition from hospital to home, potentially leading to poor patient outcomes. The Improving the Safety and Continuity Of Medicines management at Transitions of care programme included: understanding the problems faced by patients and healthcare professionals; developing and co‐designing the Medicines at Transitions of care Intervention (MaTI); a cluster randomized controlled trial testing the effectiveness of a complex behavioural MaTI aimed at improving medicines management at the interface between hospitals discharge and community care for patients with heart failure; and a process evaluation. The MaTI included a patient‐held My Medicines Toolkit; enhanced communication between the hospital and the patient's community pharmacist and increased engagement of the community pharmacist postdischarge. This paper reports on the patients' experiences of the MaTI and its implementation from the process evaluation.

**Design:**

Twenty one‐to‐one semi‐structured patient interviews from six intervention sites were conducted between November 2018 and January 2020. Data were analysed using the Framework method, involving patients as co‐analysts. Interview data were triangulated with routine trial data, the Consolidated Framework for Implementation Research and a logic model.

**Results:**

Within the hospital setting patients engaged with the toolkit according to whether staff raised awareness of the My Medicines Toolkit's importance and the time and place of its introduction. Patients' engagement with community pharmacy depended on their awareness of the community pharmacist's role, support sources and perceptions of involvement in medicines management. The toolkit's impact on patients' medicines management at home included reassurance during gaps in care, increased knowledge of medicines, enhanced ability to monitor health and seek support and supporting sharing medicines management between formal and informal care networks.

**Conclusion:**

Many patients perceived that the MaTI offered them support in their medicines management when transitioning from hospital into the community. Importantly, it can be incorporated into and built upon patients' lived experiences of heart failure. Key to its successful implementation is the quality of engagement of healthcare professionals in introducing the intervention.

**Patient or Public Contribution:**

Patients were involved in the study design, as qualitative data co‐analysts and as co‐authors.

## 1 INTRODUCTION

Heart failure is a major challenge to healthcare systems worldwide, with almost one million people living with heart failure in the United Kingdom.^1^ Treatment involves multiple evidence‐based medicines titrated to an optimal level. Without effective management, patients may have increased symptoms, reduced quality of life and increased mortality. Poor medicines management may increase avoidable hospitalization and emergency admissions, with heart failure accounting for 5% of emergency hospital admissions.^2^

Despite the global emphasis on improving medicines management, there continue to be gaps in the system when patients living with heart failure transition from hospital inpatient to home. Treatment plans may not be sufficiently communicated between health care providers.^3^ Our earlier work in four English healthcare sites identified how process and systems errors can lead to poor medicines management between hospitals and the community for patients with heart failure. Patients may lack posthospitalization follow‐up support and orientation around medicines.^4^ Enhancing communication across the hospital community transition may facilitate medicines reconciliation and encourage improved support from community pharmacists postdischarge, reducing medicines‐related discrepancies and errors and improving medicine use. A Medicines at Transitions of care Intervention (MaTI) was co‐designed to address these issues as part of the Improving the Safety and Continuity Of Medicines management at Transitions of care (ISCOMAT) programme.^5^, ^6^

The ISCOMAT programme tested the effectiveness of MaTI in a cluster randomized controlled trial (cRCT) aimed at improving medicines management between hospital discharge and community care for patients with heart failure to improve medicines use and reduce harm. A process evaluation was conducted in parallel to the trial.^7^ There is limited research on patients' experiences of interventions to improve outcomes when living with heart failure. Such insight has the potential to enhance our understanding of how interventions can be effectively implemented or may not work as intended. In this paper, we sought to understand the implementation and experience of the MaTI from the patient perspective.

## 2 METHODS

### 2.1 The ISCOMAT programme

For the cRCT, the aim was to recruit 2100 (1050 control, 1050 intervention) patients from cardiology wards in 42 National Health Service (NHS) acute trusts in England over 12 months, with a recruitment target of 50 patients per site.^8^ A process evaluation was conducted alongside the trial.^7^ In this paper, we present the findings from the process evaluation patient interviews and triangulate with the trial data on MaTI adherence.

### 2.2 The ISCOMAT Medicines at Transitions intervention

The MaTI was co‐designed with healthcare professionals and patients.^6^ It consisted of a patient‐held ‘My Medicines Toolkit’ in booklet format with four key sections: (1) My Healthcare Team, with contact details of their healthcare team; (2) My Medicines Checklist to help manage medicines; (3) Managing My Medicines, with information about the patient's medicines, side effects and how to take them and (4) Managing my Symptoms, ‘traffic lights’ to help patients monitor changes to their symptoms of worsening heart failure and know when they should seek help. Green symptoms suggest patients should keep watch, amber symptoms that they should stay alert and red symptoms that they should act, seeking appropriate support. Patients could complete a pull‐out sheet to monitor their condition, to record changes in their health, along with a medicines discharge log to be completed by hospital staff.

**Figure 1:**
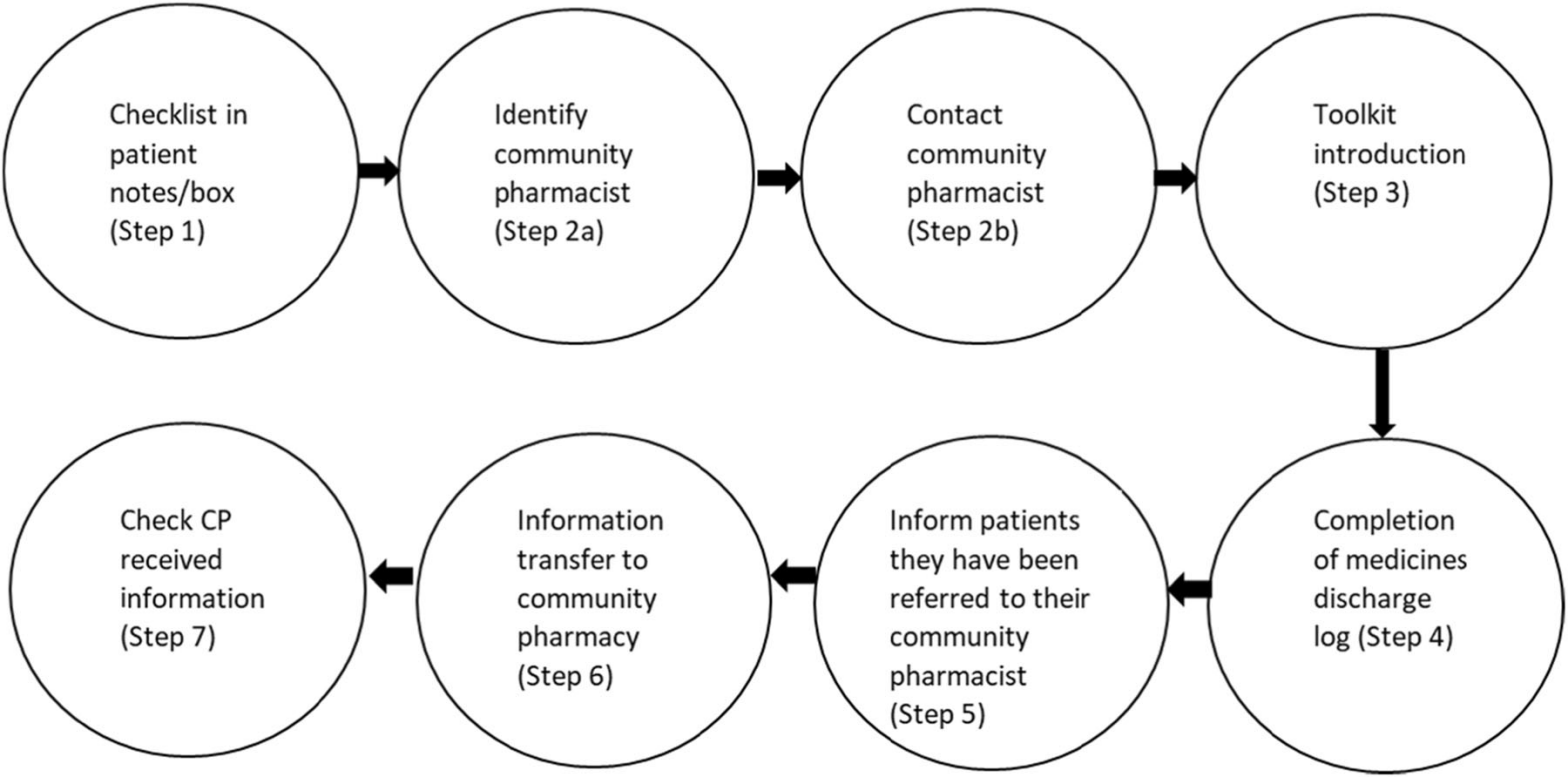
MaTI seven steps. MaTI, Medicines at Transitions of care Intervention.

The MaTI involved seven steps for hospital staff to complete, outlined in Figure 1. The hospital transferred the list of medicines on discharge to the community pharmacy to facilitate medicines reconciliation postdischarge. This could be undertaken by post, fax or electronic transfer, depending on the site's preference. Community pharmacists were also encouraged to offer a medicines discussion or Medicines Use Review (MUR) postdischarge.^9^

**Figure 2:**
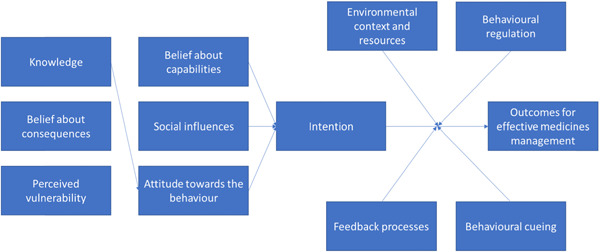
Logic model.

Once patients returned home, they could continue to consult and use the My Medicines Toolkit to support them in optimally using their medicines and managing their heart failure. Mechanisms of actions, by which behaviour change is enacted,^10^, ^11^ were considered and a logic model was developed for the toolkit (Figure 2).

### 2.3 Study design

To understand patients' experiences, we undertook an exploratory qualitative study with one‐to‐one semi‐structured interviews with trial participants and triangulated these data with routine trial adherence data to the ‘seven steps’ intervention implementation (steps indicated in Figure 1). Ethical approval was granted for ISCOMAT.

### 2.4 Sampling

Sites were purposively selected to include a range of university and non‐university hospitals, differing methods for transferring medicines discharge information to community pharmacists and covering different geographic areas of England (Table 1).

**Table 1:** Characteristics of the six process evaluation sites

Sites	University (U) or non‐university (NU)	Location	Method of transferring discharge information to community pharmacy
Site 1	NU	North East	Fax discharge/post
Site 2	U	South East	Mail
Site 3	NU	North West	Electronic transfer (PharmOutcomes®)^12^
Site 4	U	North East	Phone/NHS email/post
Site 5	NU	North West	Mail
Site 6	U	Midlands	Mail

Abbreviation: NHS, National Health Service.

Interviewed patients were recruited from the ISCOMAT cRCT. The inclusion criteria were: heart failure with evidence of at least moderate left ventricular systolic dysfunction confirmed within the last 5 years, aged 18 years or over at the time of admission, planned discharged to their own home or a care home, planned discharge to within the geographical area of that cluster and capacity to provide informed consent. Exclusion criteria were patients in a terminal phase of illness/end‐of‐life care pathway, who were not expected to survive beyond 6 weeks from the date of discharge.

A purposive sampling strategy was employed to capture patients with a range of characteristics that may have influenced their experience of the MaTI. We therefore sought to recruit 20 patients with different ages, genders, ethnicities and heart failure diagnosis length (< or >6 months) and who were patients across six different hospital sites selected for the process evaluation^7^ (see Table 1).

### 2.5 Theoretical approach

Our evaluation was informed by the Consolidated Framework for Implementation Research (CFIR), which emphasizes the need to understand whether the intervention addresses the needs and resources of participants and the engagement of innovation participants (patients).^13^

### 2.6 Data collection

Nineteen face‐to‐face interviews and one telephone interview, lasting approximately 45 min were conducted with participants between November 2018 and January 2020, approximately 3 months postdischarge. Twelve patients were interviewed within 3 months plus 1 week, four patients were interviewed within 3 months plus 1–2 weeks and four patients were interviewed 3 months plus 2 weeks post‐discharge, respectively. Waiting 3 months was necessary to allow sufficient time for patients to engage with the toolkit at home and for the community pharmacy to have the opportunity to arrange a medicines discussion with patients and to prevent influencing patient and community pharmacy behaviour, thus minimizing bias to the trial. The two researchers were both experienced qualitative interviewers and had no relationship with participants before study commencement. Potential participants were provided with an information sheet and informed consent was sought. A topic guide (Supporting Information: Appendix S1), informed by the CIFR, was developed by the process evaluation team. Interviews were audio‐recorded and transcribed verbatim by a professional transcription company.

The patient interview findings were triangulated with routine trial data on adherence to the seven steps (steps indicated in Figure 1) and from community pharmacies on whether they reconciled medicines or offered a medicines discussion/MUR, for a subsample of 124/691 17.9% of intervention patients following discharge.

### 2.7 Data analysis

We analysed process evaluation data before the analysis of trial outcomes to reduce bias in our interpretation (trial outcome data will be published in 2023). A rigorous data analysis process was conducted involving the Patient‐Led Steering Group (PLSG) as patient co‐analysts in applying the framework method.^14^, ^15^ We have previously published the method and evaluation of the co‐analysis process, which involved seven key steps: Step 1: Transcription and Anonymisation; Step2a and 2b: Familiarization with the interview; Step 3: Coding; Step 4: Developing a working analytical framework; Step 5: Applying the analytical framework; Step 6: Charting data into the Framework matrix and Step 7: Interpretation.^16^ Data were inductively coded and the analysis was manifest, based on patients' descriptions. The qualitative interview data and quantitative routine trial adherence data were consolidated through a parallel mixed analysis process. This involved independently analysing the data and integrating the data through meta‐inferences.^17^ These key findings are presented in the results section. Interpretation of the patient interview and trial data were then considered in light of theory; the CFIR^13^ and a logic model developed for the toolkit (Figure 2) are highlighted in the Section Conclusion, 4.

## 3 RESULTS

### 3.1 Recruitment process

Thirty‐six patients were iteratively approached to ensure our sample met the criteria. Reasons for nonparticipation are outlined in Supporting Information: Appendix S2.

We recruited a range of participants according to age, gender, site and length of diagnosis (Table 2).

**Table 2:** Summary of participant characteristics

Age	Number
<50	3
50–59	3
60–69	4
70–79	8
>80	1
Unknown	1
Gender	
Male	15
Female	5
Intervention cluster	
Site 1	4
Site 2	4
Site 3	3
Site 4	3
Site 5	2
Site 6	4
Diagnosis length	
<6 months	11
>6 months	7
Unknown	2

**Figure 3:**
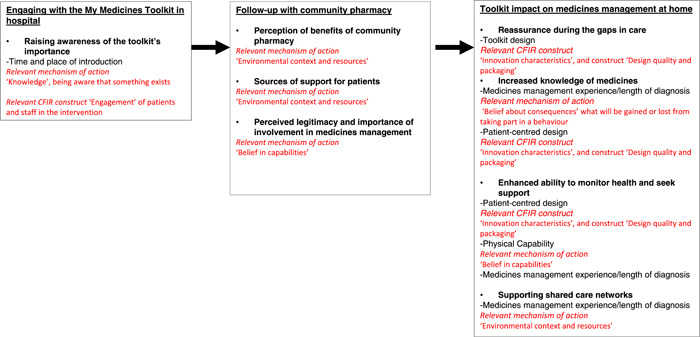
From hospital to home: Key themes and subthemes.

From the analysis, the following themes are presented as outlined in Figure 3 and explored below. Where CFIR constructs and mechanisms of action influenced themes/subthemes, these have been identified and considered.

### 3.2 Engaging with the My Medicines Toolkit in hospital

Patients first experienced the toolkit in the hospital where it was introduced to them by a member of the clinical team. This was a critical point at which hospital staff had the potential to encourage patients to engage with the toolkit. Routine trial data revealed that 18/20 interviewed patients were introduced to the MaTI by ward staff and provided with the toolkit. Of these, 14 patients had their medicines discharge log completed and located within the toolkit pull‐out sheet. Patient interview data further revealed themes on staff ‘raising awareness of the toolkit's importance’ and the ‘time and place of the toolkit's introduction’.

### 3.3 Raising awareness of the toolkit's importance

Patients became engaged with the toolkit through well‐delivered introductions, which emphasized the toolkit's importance, raising patients' awareness of the toolkit's content and importance for managing their medicines. Those who described receiving a less comprehensive introduction were less engaged. Two patients reported not receiving the toolkit. If staff did not highlight the value of the toolkit to the patient, by explaining it in detail, the patient did not engage with it, despite acknowledging that it was important. One patient described how the ward pharmacist increased their awareness, using the toolkit to explain medicines management in detail.

Nobody told me I had to take [the toolkit]…it should have been more explained, nobody explained the importance of MaTI, and it is important…I didn't really take much notice at the time until I came home. Site 4 Patient 10

We went through it all. It was very helpful…I didn't have any knowledge of any of the tablets or why I was taking them, and it was only when I spent a good hour with the pharmacist, and she explained… every tablet and what it was doing for me and up until then I didn't know. Site 3 Patient 9

### 3.4 Time and place of the toolkit's introduction

The toolkit was intended to be delivered within the hospital, and for patients to engage with the toolkit at home. This was in recognition of the fact that some patients may not be well enough to fully engage with the toolkit while in hospital but are able to engage at home. The timing of the toolkit introduction impacted patient engagement. Some patients described how the toolkit should have been delivered earlier during the hospital stay when they would have been more receptive rather than at discharge. For example, one patient was frustrated that the importance of using the toolkit had not been communicated to them due to the discussion taking place in a rushed manner at discharge.

I was on my final day and there was a big rush to get me out so it was all rushed through which may explain why I didn't realize…I think I left [the toolkit] in the hospital…It wasn't made clear to me that I was meant to take it home and use it…I would make people aware of the toolkit. Tell them that they've got to use it, read it and use it on a weekly basis and get them to read it and not just scan over it. Site 3 Patient 9

Effective communication, along with how and when the toolkit was introduced by hospital staff was crucial in raising patients' awareness about the value of the toolkit. Explaining sections within the toolkit was a key way of addressing this. However, not all patients reported the staff engaging with them in this way. Thus, staff engagement with the toolkit (a CFIR construct) was a core part of the implementation, which impacted patients' experience of the intervention. Moreover, the findings aligned with our logic model, that a key mechanism of action is ‘knowledge’, being aware that the toolkit exists.

### 3.5 Follow‐up with community pharmacy

The MaTI was designed to encourage community pharmacy to contact patients to undertake a medicines discussion or a MUR. Community pharmacy data were available for seven interviewed patients. While limited, data indicated that only two out of seven patients received an MUR, and community pharmacists reported receiving discharge information for only one of the seven patients. Interviews with patients corroborated this finding, with only one patient reporting being invited and having an MUR, and two patients having a medicines discussion. One patient was offered a medicines discussion but declined and a limited number of patients had impromptu discussions with the community pharmacy. Patients' engagement with community pharmacy is explored below.

### 3.6 Perception of benefits of community pharmacy

A limited number of patients described benefits from community pharmacist interactions. For some patients, the community pharmacist was regarded as having the necessary knowledge and a trustworthy source of support to help with medicines. Sometimes, the toolkit enhanced patients' knowledge of what a community pharmacist could offer. However, they did not always perceive benefits from the discussions. One patient described having a detailed discussion with the community pharmacist about side effects and optimum times of day to take medicines, where previously they were more accustomed to annual medication reviews with their general practitioner (GP). Other patients lacked an awareness of the community pharmacist role and were sceptical about whether they would be willing or able to help, despite the explanation provided in the toolkit. A key mechanism of the action noted was the environmental context (of community pharmacy) and resources.

[The toolkit] gave me the idea what's available once you've left hospital and that you can ask a lot more questions about your pharmacist rather than going to your doctor all the time. [The medicines discussion] didn't have any lasting impression on me…I don't think I got any benefit. Site 5 Patient 11

Most people don't know…[I] never knew that that's what the pharmacist does is advise people. Site 1 Patient 4

### 3.7 Sources of support for patients

Community pharmacist support was occasionally rejected where existing or additional sources of support were working well for patients. For example, existing community healthcare support and resources in the form of community heart failure clinics were accessed and considered effective and sufficient by some patients. A key mechanism of the action noted was the environmental context (of community pharmacy) and resources.

Within the last month my pharmacy phoned up…they offered advice if I wanted it, but I didn't take it because obviously I'd been going to the…outpatients centre…the primary care cardiac failure centre… I go every couple or three months. Site 1 Patient 5

### 3.8 Perceived legitimacy and importance of involvement in medicines management

Perspectives on who should legitimately be involved in medicines management influenced patients' experiences. Patients sometimes perceived a hierarchical structure among health professionals with one patient describing how they would only take notice of advice from their GP. Some patients felt they should have minimal involvement in their medicines management, assuming that their own experience was less valid than that of health care professionals. This aligned with our logic model, which mapped how patients may not take action if they lack belief in their capabilities (a key mechanism of action being belief in capabilities). In addition, some patients preferred a more reactive than preventative approach given the burden of having to take multiple medicines.

If the doctor, tells me to do something, no matter what anybody else says, [I only pay attention to the doctor]…[The doctor] knows more than I do. Site 1 Patient 1

I'm no judge of whether it's serious or not, I don't know whether I would look at it after that. Site 1 Patient 2

I just take what they've given me…I've got that many tablets… I'm not one of those who [bothers]… when you get new prescriptions… I've got a couple of friends; they go straight to the side effects…I don't… I take them, and if I'm not very well, I look to see if [the new medicine] could be [the cause]. Site 6 Patient 20

Thus, the apparently limited impact of the intervention on community pharmacist engagement led to some patients not having an increased level of interaction and support around their medicines management. This lack of engagement was compounded by patients' perceptions about managing medicines and the community pharmacist's role in their care. Perceptions that other sources of support were more reliable meant that patients' experiences with community pharmacy were limited.

### 3.9 Toolkit impact on medicines management at home

Patients' engagement with the toolkit at home varied. Nine of the twenty patients used the toolkit at home as intended, drawing on information when it was important to them. Six patients had limited engagement, using it only briefly when they received it in hospital, or shortly after and had not revisited it since. Five patients had no engagement with the toolkit at any stage of their transition in care.

The four key themes below explore how the toolkit may have enhanced medicines management for some patients while having a limited impact on others.

### 3.10 Reassurance during the gaps in care

Once patients were discharged from the hospital they returned home with a copy of the toolkit in most cases. The toolkit offered reassurance during the transition, until community healthcare support, such as the community heart failure nurse was available, and as a source of ongoing support between visits to health care professionals (community heart failure nurses, GPs). For these patients, wellbeing was enhanced by giving them confidence that there was a good quality source of support (toolkit) they could draw on until they received further help. One patient felt the toolkit demonstrated compassion towards people living with heart failure. The toolkit provided reassurance in between health care professional visits within the community and became a simple, trusted point of reference.

You can never have enough information…it does help, it's a bit of reassurance, it's an object that someone cares. Site 4 Patient 19

Realistically you don't need the [toolkit] because you can phone, but the [toolkit]…they're nice to have because after a certain time you can look, hang on, what did she say. Site 4 Patient 19

### 3.11 Increased knowledge of medicines

Learning about medicines was a key reason patients engaged with the toolkit at home. It improved their knowledge of what medicines do and their side effects. This new knowledge had important implications for how patients managed their medicines and adherence to medicines regimen was potentially improved through patients' increased understanding of their medicines. This patient described how they began to take more notice of how medicines should be taken and became committed to doing so.

This booklet is like a bible; it explains everything to me…it's only through reading the instructions on all the drugs that I became aware of what the tablets were doing to me and then I started to take them more regularly. Site 3 Patient 9

The toolkit enabled some patients to be more active and informed in their own medicines management, as opposed to passively taking medicines as instructed. Both newly diagnosed and patients with longer‐term heart failure diagnoses benefited from the toolkit increasing their knowledge. Some had established management systems, yet the toolkit further enhanced their knowledge of medicines. Such patients were interested in learning the detail of why they were taking certain medicines and the side effects (links to the mechanism of action belief about consequences). On the other hand, there were patients with long‐term heart failure diagnoses who felt there was limited new information for them, although the toolkit could still be used as a reference aid.

I thought I knew what the medicines were for, but they did explain a couple of other things about the medicines that I didn't know, side effects. Site 4 Patient 17

I know why I'm taking them…Whereas, in the past…I was just munching them down every morning, noon and night and just doing as I was told. Site 3 Patient 9

I read it when they gave it to me in hospital… I don't think [I learnt anything new from the ‘my medicines section’]. Site 6 Patient 20

Some newly diagnosed patients felt too distressed or unwell to engage with the toolkit. Patients could be overwhelmed by their diagnosis and needed time to adjust, not only in the hospital but once they had returned home. The toolkit design enabled patients to ‘dip in’ to understand medicines and was highly regarded by most patients. The design thus facilitated patients' ability to learn more about their medicines and side effects. For some older patients, the design of the toolkit was inaccessible due to the size of the print.

She did [explain how to use the toolkit and talk through it], but…It's… Just me…I'm very, very lethargic at the moment and it's just my health. Site 3 Patient 15

I thought the layout was good…the side effects…If I started to get an irritating cough for example…I'd pick the book up and go through it to see if it's related to my tablets. Site 1 Patient 18

### 3.12 Enhanced ability to monitor health and seek support

With the final toolkit section, ‘My symptoms’, many patients reported an enhanced ability to monitor their heart failure. For example, in Site 3, a patient was prompted to check for signs every day. The design of the pull‐out sheet supported patients who were more able to embed monitoring in their daily lifestyle when they needed it. Patients described how drawing on this section was helpful in the beginning and once they were aware of their medicines, the pull‐out sheet was no longer needed (links to the mechanism of action belief about capabilities). For others, the pull‐out sheet was unhelpful and could become easily lost. One patient described how the monitoring section of the toolkit could not be used due to physical difficulties in taking the required measurements.

When I immediately came out of hospital, that was what I was using as a reference. I put my tablets up once a week and I use that list, but I know what I'm using now. Site 4 Patient 18

I have that much wrong with me…I'd probably fill it in in one go…What is my blood pressure‐ I don't know? What is my weight‐ I can't get on the scales…Are my legs more swollen than normal? ‐ No, they're [prosthetic]. Site 4 Patient 18

Patients in some cases described how the toolkit would prompt them to seek support when appropriate.

I'd pick the book up and go through it to see if it's related to my tablets and then I'd make a phone call, rather than going straight to my GP… It's a good reference book. Site 2 Patient 5

Patients who had lived with heart failure for many years had pre‐existing processes in place to monitor their health changes and had been previously provided with a lot of information. Thus, while they were often motivated to manage their medicines, they found the toolkit less relevant as they had established monitoring strategies.

### 3.13 Supporting shared care networks

Some patients had networks of informal care such as family, and formal care professionals, such as community heart failure nurses, to help them manage their medicines. The toolkit could support these partnerships. The impacts were enhancing patient and family knowledge of medicines, and enhancing communication between professionals, family and the patient. Shared care could help where patients were less able to engage, with examples described of how patients' medicines management was shared between professionals, family and themselves, and how the toolkit supported this process. The toolkit also enhanced family carers' understanding of heart failure and related medicines management. For example, a patient who had difficulty engaging described how their wife was able to learn about side effects and recognize these as important. Another patient described how the toolkit could have helped them explain their medicine management practices to work colleagues, and in the case of emergencies, inform first responders.

When I first got it [the toolkit], I had to get my aunty and uncle to read it because… it's a lot to take in and they're more academic than me… they'll explain it in case when [the heart failure nurse] has said anything, if it's forgot or I've misheard, it's like a double reminder. Site 4 Patient 19

[My wife's] got an idea…I was getting really cold, which I've never been like that, and she said ‘no, that's probably down to your medication…that's one of your side effects’…So she's read it and digested it more than me. Site 1 Patient 3

Another patient described ‘I took in my discharge letter…it has my medication and what's actually gone on, in case anything happens at work, and they have to call an ambulance…They have that to show anyone attending…I should've taken [the toolkit] to work and let…everyone have a read’. Site 1 Patient 3

Where existing monitoring strategies involving formal and informal care networks were felt to be effective, this could negate the need for the toolkit. In the following, a patient described a formal and informal care network for identifying and communicating symptoms.

I've dealt with the problem for that long that I know the signs…I'll say to [my wife], if I've washed, ‘I've got more breathless this morning’, or, ‘I've put a bit more weight on’, or something. I'll tell [my wife] and if she notices that I am a bit more breathless she'll say so, and then I can tell the nurse’. Site 6 Patient 17

Medicines management was therefore perceived to have been enhanced for some patients through the use of the toolkit at home. The toolkit was incorporated into the daily lived experience of managing heart failure medicines leading to many patients valuing the toolkit as a source of support (links to the mechanism of action environmental context and resources). The content of the toolkit, facilitated by its design and access at home (relates to CFIR constructs Innovation characteristics and Design quality and packaging), was valued by some patients as it enhanced their knowledge of medicines, how they monitored medicines and supported their care networks.

## 4 DISCUSSION

Care transitions between hospitals and the community can be fraught with challenges impacting patients' experiences of medicines management.^18^, ^19^, ^20^ The MaTI was co‐designed to improve such transitions by enhancing patients' understanding of their medicines, their treatment, how to manage side effects, how to monitor their condition and healthcare support including community pharmacy follow‐up.^6^ We, therefore, sought to explore the implementation and experience of the MaTI from the patient's perspective.

Patients' experiences of the MaTI were largely related to the toolkit. We identified themes across the patients' transition from ‘engaging with the My Medicines Toolkit in hospital’, to ‘medicines management at home’ and having a ‘follow‐up with community’. Patients' ‘perceptions of the benefits of community pharmacy’ impacted their experiences. Patients sometimes opted for support other than the community pharmacist, particularly where others were regarded as having a more important or legitimate role.

A limited number of themes identified map onto the CFIR,^13^ which we have applied as our overall theoretical approach to the process evaluation. CFIR domain ‘Innovation characteristics’, and construct ‘Design quality and packaging’ considers whether the intervention is perceived to be presented and assembled well as this can either facilitate or be a barrier to patients' engagement. For example, when a patient perceived the toolkit to be well designed, they were able to increase their medicines knowledge. Most patients felt the toolkit was well designed, although a minority felt the text could have been enlarged. The co‐design approach to intervention development, which involved patients and staff, was instrumental in achieving this patient‐centred design.^6^

The CFIR construct ‘engagement’ was important on two accounts, first, some hospital staff were described as more engaged in the delivery of the toolkit than others; and second, we found that patient (CFIR sub construct ‘innovation participants’) engagement was influenced by staff delivery of the toolkit in hospital. Patients' engagement varied according to physical capability and experience in medicines management/length of diagnosis.^13^ Thus, facilitators and barriers to implementation included the toolkit design itself, which in most cases facilitated the implementation of the intervention; however, it could be a barrier, for example, where a minority of patients felt the font was too small. Where staff emphasized the importance of the toolkit at the right time and place, this facilitated implementation. Mechanisms of actions^10^, ^11^ were considered as this allows a range of potential influences on behaviour to be examined, and the logic model developed for the toolkit was retrospectively applied to the results. Mechanisms included ‘Knowledge’, being aware that something exists, ‘belief about consequences’, ‘environmental context and resources’, ‘beliefs about capabilities’ and ‘attitude towards behaviour’. For example, in the case of the latter, patients had preconceptions about healthcare professional roles and their own roles in their healthcare. Some patients lacked ‘beliefs about capabilities’ to become involved in their own medicines management as they felt healthcare professionals had all the expertize required.

Despite the MaTI being designed to encourage greater interaction between community pharmacy and patients, limited interaction took place. Some patients' perceptions of the benefits of community pharmacy were also relatively limited and they were often unaware of the services offered despite the toolkit seeking to increase patients' knowledge of community pharmacy services. Patients' lack of understanding of community pharmacy appears to be a widespread issue with a systematic review of patient views of community pharmacies demonstrating low awareness of roles.^21^ Patients were often unaware of the range of services on offer, regarding the pharmacy as a dispensary, place to purchase medicines or discuss minor ailments. One study found that few patients were aware of MURs and the New Medicines Service.^22^ A lack of understanding was associated with low take‐up of services. To increase awareness, building on convenience and relationships between pharmacists and patients was recommended.^21^ Moreover, some patients may not engage with community pharmacy due to their way of managing medicines. A typology of patient self‐management of medicines for chronic conditions found that patients may be ‘Self‐determined and highly self‐managing’, seeking information from multiple sources, ‘Security‐seeking and self‐managing’ enhancing knowledge already received from health professionals, ‘Dependent with limited self‐managing’ relying more on discussions with health professionals, or ‘Co‐managing with close family’ where the family member is a source of information.^23^ Our findings indicated that some patients could have combinations of such types, for example, comanaging with close family while also being self‐determined to self‐manage, and using resources such as the toolkit, and having discussions with health care professionals.

### 4.1 Implications for research, strengths and limitations

The key strengths included our methods in both co‐designing^6^ and co‐evaluating^16^ the intervention with patients, which brought us closer to understanding and incorporating patients' experiences. An experience‐based co‐design approach was applied to intervention development. Our analysis was greatly enhanced through the involvement of our ISCOMAT PLSG as co‐analysts. The PLSG provided vital context to data which they were able to do through knowledge acquired through lived experiences of heart failure. PLSG perspectives on the data thus enhanced the validity and rigour of our data analysis and interpretation.^16^

There were limitations to our data collection. Some patients had difficulty recalling details of the toolkit due to the time that had elapsed since last engaging with it and their illness in the hospital. However, this was necessary to ensure there was sufficient time for community pharmacists to have medicines discussions or MURs with patients, for patients to engage with the toolkit at home and to minimize bias during the trial. The analysis was based on interviews at one single time point and a longitudinal approach may have indicated changes in patients' experiences and the processes underpinning these. Community pharmacy data were only available for seven interviewed patients, therefore for the remaining patients we were solely reliant on their accounts, and it is possible that patients were unaware if they had received an MUR or not.

The sampling frame was limited to patients who had consented to take part in the cRCT. While it was our intention to have an ethnically diverse sample, at the time of sample selection, there was no ethnic diversity in the sampling frame. We originally intended to sample participants based on adherence; however, this was not possible due to the delayed return of data. Therefore, further evidence could support our understanding of whether the intervention is feasible and acceptable to a broad range of participants. We were however able to recruit patients from all our six process evaluation sites, and with differing age, gender and length of diagnosis. The latter was particularly important for revealing differing experiences of MaTI.

#### 4.1.1 Research, policy and practice implications

Our findings indicated that while the toolkit was delivered to 18 out of our 20 participants, the quality of this delivery varied. We have shown that the quality or lack of implementation of the intervention has implications for patients' experiences. Patient experience with community pharmacy was not significantly enhanced, with patients hesitant to engage, preferring other sources of support such as the community heart failure nurse. However, the toolkit did enhance some patients' experience with medicines management. Implementation and roll out of the intervention varied according to personal resources such as wellness and length of diagnosis. Moreover, there was evidence to suggest that some patients' attitudes to medicines management altered because of the intervention. This may have implications for primary care in terms of reducing GP workload, and potentially reducing avoidable hospital admissions as patients could avoid deterioration in their own health.

There have been changes in the NHS since ISCOMAT began with the Medicines Discharge Service being introduced in February 2021 to improve communication between community pharmacy and hospitals.^24^ Within this new context, patients may have improved experience with their medicines management. The findings from this paper suggest that such communication gaps need addressing, and a toolkit could potentially facilitate the transition process. Moreover, the policy now emphasizes encouraging patients to self‐manage^25^, ^26^ and there have been advances in this approach.^27^ However, in practice some patients may not feel that they should or can be involved, perhaps, particularly those who have lived with heart failure for many years, before these advances. Further research such as a Phase 2 explanatory trial could test these mechanisms and the logic model. Once the trial outcomes are known we will further contextualize our findings.

## 5 CONCLUSION

The MaTI intervention has the potential to enhance patients' lived experiences of medicines management, building on their existing support networks, improving their ability to monitor and seek support and increasing their knowledge of medicines. Patients felt that the key to this was the engagement of healthcare professionals in implementing the intervention, raising patients' awareness of its importance. The MaTI intervention demonstrates the potential benefit of this type of tool in supporting patients with heart failure to improve medicines management across the hospital/home transition. The patient experience of its use provides critical insights into its value to better support patient care, with the aim of improving patient experience and outcomes.

## AUTHOR CONTRIBUTIONS

All authors revised the manuscript critically for important intellectual content and made substantial contributions to conception and design. Catherine Powell drafted the manuscript. All authors have given final approval for the version to be published. Each author has participated sufficiently in the work to take public responsibility for appropriate portions of the content and agreed to be accountable for all aspects of the work in ensuring that questions related to the accuracy or integrity of any part of the work are appropriately investigated and resolved.

## CONFLICT OF INTEREST

The authors declare no conflict of interest.

## ETHICS STATEMENT

Ethical approval was granted by the Health Research Authority REC: 18/YH/0017/IRAS: 231431.

## Supporting information

Supporting information.

Supporting information.

## Data Availability

The data that support the findings of this study are available on request from the corresponding author. The data are not publicly available due to privacy or ethical restrictions.
