# Supplementary material for: Experiences of patients with heart failure with medicines at transition intervention: Findings from the process evaluation of the Improving the Safety and Continuity of Medicines management at Transitions of care (ISCOMAT) programme
Source: Health Expect. 2022 Jul 31;25(5):2503–14. doi: 10.1111/hex.13570 (PMC9615069; doi:10.1111/hex.13570)
Supplement: Supplementary file 1 — Supporting information. [file HEX-25--s002.docx]

**APPENDICES**

**Appendix 1 Topic Guide**

**ISCOMAT Process Evaluation - Patient interview schedule**

Briefing- Permission to record.

Thank you for agreeing to be interviewed. I would like to ask you some questions about your experiences of using the ISCOMAT toolkit. There are no right or wrong answers – I am interested in your opinions and experiences.

1. **Receiving the My Medicines Toolkit**

- Do you remember receiving the ‘My Medicines Toolkit (show an example?)
- Can you remember who gave you the ‘My medicines’ toolkit while you were in hospital HFSN/Pharmacist/ Other?
- Did they explain how to use the toolkit? (Why you were given it? / What you should use it for?)
- Can I have a look at your copy of the toolkit?
- Did the hospital complete the section called ‘My heart failure medicines’?
- Did they explain how to complete the sections? Which sections you should complete/when?
- Was it explained that you should take the My Medicines Toolkit along to ALL your healthcare appointments?
- Did they explain what to do if you did not understand anything in the My Medicines Toolkit?
- Did they give you the opportunity to ask questions after receiving the My Medicines Toolkit? If yes, what did you ask?

1. **Using the toolkit**

- Once at home how often did you use the My Medicines Toolkit?
- If they did not use the toolkit- What was the main reason that you did not use the toolkit?
- Too difficult to understand?
- Didn’t feel confident in completing it?
- They didn’t know the answers?
- Any other reason?
- Did your relative/carer look at the My Medicines Toolkit? If so, what did they think about it?
- Was it explained to you that the checklists are to be completed by you once at home?
- Did you manage to fill in the checklists (my medicines)? If not, why?
- Too difficult to understand (any sections in particular)?
- They did not know the answers (probe- which questions?) • Any other reason?
- What is your view of the traffic light symptom checker? How useful did you find it?
- What were your overall views of the My Medicines Toolkit? (Easy to understand/ use or difficult) If so why?
- Is there anything in particular that you liked/disliked about the toolkit?
- Is there anything that is not in the My Medicines Toolkit that you would like to see added/ taken away?
- Did the My Medicines Toolkit contain information that you found useful? If so, can you give examples?
- Did it contain any information that was not relevant to you? If so, can you give examples?
- What do you think of the amount of information in the My Medicines Toolkit? Is it too long/short?
- How could the My Medicines Toolkit be improved? (Probe: design/ ease of use/ content)
- Did you show your toolkit to any other healthcare professional? For example, your GP or community heart failure nurse.
- If yes, what did you show them? What did you discuss? Did it help you and them to discuss your medicines?

1. **Overall impressions of the toolkit**

- Did the My Medicines Toolkit help you?
- Manage/ understand your medicines?
- Understand more about your condition?
- Help you when speaking to your healthcare team about your condition/medicines?
- Would you use it in the future?
- Would you like to see similar toolkits for other conditions you may have?
- What were the challenges you faced in using the My Medicines Toolkit?
- Would you be willing to use an electronic (mobile phone app) version of the My Medicines Toolkit?
- Would you like to see the My Medicines Toolkit in other formats (electronic- mobile phone app/ film format- role-play between patient/ hps)?
- Would you recommend this type of ‘toolkit’ to a friend/relative with a similar condition?

1. **Contact with your local/ community/ practice Pharmacist**

- Do you have a regular community pharmacy that you use?
- Tell me about your relationship with your community pharmacist?
- Have you previously sought advice from your community pharmacy about medicines or other health concerns you may have?
- Were you contacted by your community pharmacist after you left hospital? How were you contacted? What did they contact you about?
- When you left hospital, the hospital sent your list of discharge medicines to your community pharmacist – did they talk to you about this? Do you know if they did anything with this list?
- Did the pharmacist offer to speak to you about your medicines or review your medicines, or do something called a Medicines Use Review*? If so, did you take this up? If so, what happened? What did the pharmacist do? What did you discuss? did you find the review useful? In what way? **[*Important to establish whether a formal Medicines Use Review was undertaken, or if not, a medicines discussion*].**

**may need to explain what a Medicines Use Review involves to jog memory/clarify i.e. invited by pharmacist to discuss medicines, pharmacist will invite you into consultation room, pharmacist will take consent, discussion around medicines, paperwork completed, GP informed if an issue found*

If no, why not. Probe for reasons here – housebound, time, access, didn’t see point etc

**Do not encourage patient to ask for MUR if they have not attended**

- Manage/ understand your medicines?
- Understand more about your medicines?
- Have you ever seen a pharmacist in your GP practice? If yes can you tell me what they discussed with you?
- If we were to roll out the My Medicines Toolkit across the country, how do you think we should do this?
- What might we have to change/adapt?
- What you think the problems might be? How could we overcome the problems?
- Is there anything we should do differently in the hospital when patients are being discharged?
- What about the community pharmacist? Is there anything we would need to change/adapt from that perspective?

1. **Now I would like to ask you about your experiences with your medicines since leaving hospital?**

- How many different medicines do you now take? What are they for? At what times of day do you take them?
- How easy or difficult has it been to take your medicines as instructed? Why is this the case?

1. **How confident are you taking your medicines?**

- Are you taking them? To what extent do you feel they are effective? Do you feel safe taking them? Why / why not? Do you feel like they help your condition(s)?
- Has anything happened since you left hospital to make you more or less confident in your medicines?
- How much do you understand about how and when to take your medicines? And why you are taking them?

**Thank you. Debrief. Do you have any questions?**
