# Supplementary figures and images for: Experiences of patients with heart failure with medicines at transition intervention: Findings from the process evaluation of the Improving the Safety and Continuity of Medicines management at Transitions of care (ISCOMAT) programme
Source: Health Expect. 2022 Jul 31;25(5):2503–14. doi: 10.1111/hex.13570 (PMC9615069; doi:10.1111/hex.13570)

**Appendix 2 Recruitment process**


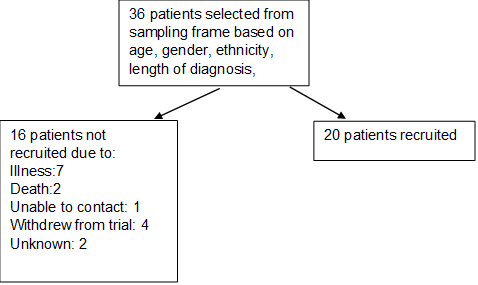

Supplement: Supplementary file 2 — Supporting information. [file HEX-25--s001.docx]
